# Plasticity first: molecular signatures of a complex morphological trait in filamentous cyanobacteria

**DOI:** 10.1186/s12862-017-1053-5

**Published:** 2017-08-31

**Authors:** Robin Koch, Anne Kupczok, Karina Stucken, Judith Ilhan, Katrin Hammerschmidt, Tal Dagan

**Affiliations:** 1 Institute of General Microbiology, Kiel University, Kiel, Germany; 2 Present address: Instituto de Investigación Multidisciplinario en Ciencia y Tecnología, Universidad de La Serena, Av. Raúl Bitrán 1305, 1720010 La Serena, Chile

**Keywords:** Cyanobacterial evolution, True-branching, Comparative transcriptomics, Genetic assimilation

## Abstract

**Background:**

Filamentous cyanobacteria that differentiate multiple cell types are considered the peak of prokaryotic complexity and their evolution has been studied in the context of multicellularity origins. Species that form true-branching filaments exemplify the most complex cyanobacteria. However, the mechanisms underlying the true-branching morphology remain poorly understood despite of several investigations that focused on the identification of novel genes or pathways. An alternative route for the evolution of novel traits is based on existing phenotypic plasticity. According to that scenario – termed genetic assimilation – the fixation of a novel phenotype precedes the fixation of the genotype.

**Results:**

Here we show that the evolution of transcriptional regulatory elements constitutes a major mechanism for the evolution of new traits. We found that supplementation with sucrose reconstitutes the ancestral branchless phenotype of two true-branching *Fischerella* species and compared the transcription start sites (TSSs) between the two phenotypic states. Our analysis uncovers several orthologous TSSs whose transcription level is correlated with the true-branching phenotype. These TSSs are found in genes that encode components of the septosome and elongasome (e.g., *fraC* and *mreB*).

**Conclusions:**

The concept of genetic assimilation supplies a tenable explanation for the evolution of novel traits but testing its feasibility is hindered by the inability to recreate and study the evolution of present-day traits. We present a novel approach to examine transcription data for the plasticity first route and provide evidence for its occurrence during the evolution of complex colony morphology in true-branching cyanobacteria. Our results reveal a route for evolution of the true-branching phenotype in cyanobacteria via modification of the transcription level of pre-existing genes. Our study supplies evidence for the ‘plasticity-first’ hypothesis and highlights the importance of transcriptional regulation in the evolution of novel traits.

**Electronic supplementary material:**

The online version of this article (doi:10.1186/s12862-017-1053-5) contains supplementary material, which is available to authorized users.

## Background

Explaining the evolution of novel phenotypic traits has been a long-standing challenge in biology [1]. A major conundrum is whether the origin of a novel trait can evolve via adaptive genetic change alone or whether it could be initiated by a plastic phenotype that is induced in direct response to an environmental cue. Phenotypic plasticity may lead to an initially suboptimal adapted organism in a novel environment, but results in survival of individuals and so enables trait refinement by subsequent natural selection [2]. This evolutionary scenario, where a primarily plastic phenotype becomes genetically encoded has been termed genetic assimilation [3]. In this scenario no changes in the genome are required for the origin of a trait as selection is acting on a phenotype that is based on the existing genetic repertoire [4]. Despite of the fact that the ‘plasticity-first’ hypothesis has been postulated long ago, there has been difficulty in providing evidence from natural populations [5, 6]. One major challenge is that once a trait has evolved, its evolution cannot be identically recreated and followed under the original conditions [6]. The best solution so far has been to perform experiments with extant lineages as ancestral-proxies to the lineage with the trait of interest [6, 7]. Here we propose to make use of the traces of past evolution in genomes of contemporary organisms by searching for molecular signatures of the ‘plasticity-first’ hypothesis for the trait of interest. According to the ‘plasticity-first’ hypothesis, one does not expect novel traits to be based on gene gain or loss but to be based on a changed expression pattern of genes; for example, a change in gene transcriptional regulation. We test this proposal by investigating the underlying genetic mechanisms of the complex trait ‘true-branching’ in a prokaryotic system – the cyanobacteria.

Cyanobacteria are classified into a monophyletic phylum that includes genera presenting a wide range of phenotypic diversity, some of which are considered a peak in prokaryotic complexity. Several species are multicellular and differentiate particular cell types, such as heterocysts, which are specialized cells that fix atmospheric nitrogen under aerobic conditions (subsection IV and V) [8]. The highest complexity is observed in species where the cells divide in more than one plane to form true-branching or multiseriate filaments (subsection V) [9]. Phylogenetic reconstructions of cyanobacterial evolutionary history show that heterocystous, filamentous cyanobacteria of the Nostocales/ Stigonematales orders (subsections IV and V) constitute a monophyletic clade [10, 11]. This indicates that the origin of differentiated heterocysts was singular. Furthermore, species forming true-branching or multiseriate filaments (e.g., *Fischerella* or *Chlorogloeopsis*) constitute a monophyletic clade within the Nostocales clade [10, 11]. This suggests that the true-branching cyanobacteria had a filamentous, branchless ancestor and that the complex colony morphology appeared later in evolution. While cell differentiation and intercellular communication has been extensively studied in branchless species (e.g., *Nostoc* and *Anabaena*) [8, 12, 13], much less is known about the mechanisms underlying the complex true-branching colony morphology.

The accumulation of completely sequenced cyanobacterial genomes has enabled a large-scale survey of genes whose presence/absence pattern is correlated with cyanobacterial morphological diversity. However, studies focusing on the identification of genes that are specific to true-branching cyanobacteria did not reveal clear candidate genes whose gain or loss could be linked to the true-branching trait [10, 11]. Representative true-branching genomes were found to be enriched in genes that function in signal transduction and transcription-related functional categories [10], though those could not be directly linked to the true-branching phenotype. The lack of genes that could be directly linked to the true-branching phenotype suggests that this trait may still hinge upon differences in the transcriptional regulation of certain genes rather than differential gene content.

Here we aim to identify the underlying mechanisms of the true-branching phenotype in cyanobacteria, focusing on the true-branching species *F. muscicola* PCC 7414 and *F. thermalis* PCC 7521. The two species are phenotypically identical and *F. muscicola* is denoted as the reference strain of the genus [9]. Nonetheless, their genotypes are very different with the *F. muscicola* genome including 7167 protein-coding genes and *F. thermalis* genome including only 5340 protein-coding genes [11]. We identify conditions in which a filamentous, branchless phenotype can be induced in *Fischerella*. The branchless phenotype represents an ancestor-like, plastic phenotype of this species. Using a novel approach that combines dRNA-seq [14, 15] with comparative genomics, we search for molecular signatures of the ‘plasticity-first’ hypothesis; those are genes whose transcriptional regulation differs between the branchless and true-branching phenotypes. Using dRNA-seq, we identify transcription start sites (TSSs) that correspond to either mRNAs or regulatory RNAs (i.e., small RNAs) and quantify their transcription level. The comparison of TSS loci between two *Fischerella* species enables the comparison of the transcriptional regulation regime between orthologous TSSs. We expect that the putative components of the true-branching morphology are regulated by conserved TSSs. The differential transcriptional regulation between the true-branching and branchless phenotypes is thus expected to be consistent between the two compared species.

## Results

### Phenotypic plasticity of the true-branching colony morphology

**Fig. 1:**
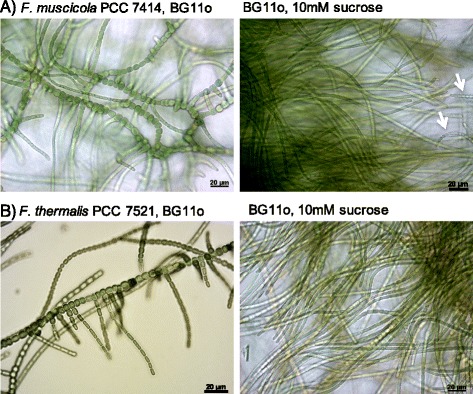
Phenotypic plasticity of colony morphology in *Fischerella*. Growth stages of *Fischerella muscicola* PCC 7414 (**a**), and *Fischerella thermalis* PCC 7521 (**b**). When grown in BG11o, *Fischerella* species show the true-branching phenotype (left panel), whereas the culture under 10 mM sucrose resulted in non-branched filaments (right panel). Hormogonia are marked with white arrows. All cultures were grown at 37 °C and a light intensity of 30 μmol/m^**2**^ s^**1**^

To screen for conditions that generate an ancestor-like filamentous and branchless phenotype, we cultured the true-branching cyanobacteria *F. muscicola* PCC 7414 and *F. thermalis* PCC 7521 under different conditions. Those included photoheterotrophic growth, heterotrophic growth in solid media, and photoautotrophic growth supplemented with NaCl. *F. muscicola* PCC 7414 and *F. thermalis* PCC 7521 are facultative photoheterotrophs and can grow on sucrose [9]. Only photoheterotrophic growth conditions (10 mM sucrose) led to cultures where branchless filaments dominated, though hormogonia could be observed as well (Fig. 1). Incubation for prolonged time spans resulted in the release of hormogonia from the main filaments and their maturation into young trichomes with heterocysts after 75 days. The effect of sucrose supplementation on cyanobacteria cell development has been so far studied in the context of cyanobacterial symbioses with plants and fungi. In *Nostoc punctiforme*, the importance of sucrose has been highlighted by recent findings suggesting that it serves as a hormogonium-repressing-factor (HRF) [16]. Supplementing the culture media with NaCl in our experiments resulted in highly branched filaments, where the main filaments also turned multiseriate (Additional file 1: Figure S1). At the tested concentrations, NaCl did not influence the growth of *F. muscicola* whereas *F. thermalis* grew slower than under non-supplemented media. We also generated the ancestral-like, aseriate phenotype of *Chlorogloeopsis fritschii* PCC 6912 and processed the samples identically to the *Fischerella* samples (for details see Additional files 1: Supplementary text).

### The primary transcriptomes of *F. muscicola* and *F. thermalis*

To identify genes whose transcriptional regulation correlates with the true-branching morphology in the two *Fischerella* species, we compared the transcriptome of cultures with the filamentous branchless phenotype to cultures with the true-branching phenotype. Transcriptional start sites (TSSs) were identified using the 5′ differential RNA sequencing approach (5′-dRNA-seq). This approach enables the quantification of transcript level and the distinction between primary and processed transcripts (for review see [17]). Resulting sequence reads were mapped to the respective genome and TSSpredator [18] was used to infer TSSs from the normalized read coverage information. TSSs occurring in a distance of ±35 bp were clustered into a single TSS. The TSSs were classified according to their position relative to open reading frames (ORFs) into genic (gTSS), antisense (aTSS), internal (iTSS) and intergenic (nTSS). The TSS annotation was further validated by testing for conserved sequence motifs that are typical for the −10-box (Pribnow box) using the MEME software suite [19]. Sequence motifs at the TSS loci confirm the presence of a Pribnow box in all TSS classes and species (Additional file 1: Figure S2).

**Fig. 2:**
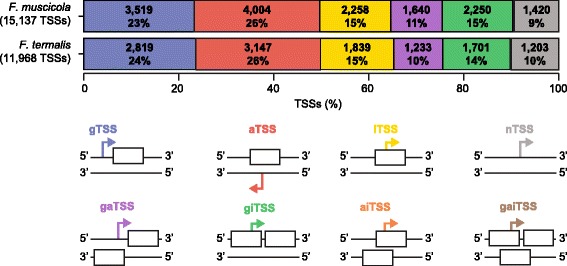
TSS class distribution. TSSs are classified according to their location relative to an open reading frame (ORF) into genic (gTSS, blue), antisense (aTSS, red), internal (iTSS, yellow) and intergenic (nTSS, grey). Depending on the complexity of the genomic region, TSSs can be classified into multiple TSS classes and are named as genic-antisense (gaTSS, purple), genic-internal (giTSS, green), antisense-internal (aiTSS, orange) or genic-antisense-internal (gaiTSS, brown). The proportions of aiTSSs and gaiTSSs are found to be very small (< 1%) and are not visible as bars

The TSS class distribution is similar between the two species (Fig. 2). On average, 26% of the TSSs fit into more than one TSS class, most of them are genic TSSs that are also internal or antisense (Fig. 2). The proportion of TSSs observed in both phenotypes is 91% in *F. muscicola* and 96% in *F. thermalis* (Additional file 2: Table S1A). The untranslated regulatory region (UTR) length distribution is similar between the two species (mean UTR length about 380, Additional file 1: Figure S3). TSSs were recovered for most of the ORFs (84% in *F. muscicola* and 86% in *F. thermalis*). For the majority of ORFs, TSSs of more than one TSS class have been detected (Additional file 1: Figure S4). The median TSS frequency per ORF is 1 for most TSS classes (Additional file 2: Table S1B). The ORF length and the frequency of TSSs per ORF are positively correlated for aTSSs and iTSSs (Additional file 2: Table S1B).

### Evolutionary conservation of transcriptional start sites in orthologous genes

The comparison of TSSs among closely related species enables the inference of orthologous and lineage-specific (i.e., singleton) TSSs. The genomic comparison between *F. muscicola* and *F. thermalis* yields a total of 2805 orthologous gene pairs. Of those, 55% contain gTSSs, 45% contain aTSSs, and 38% contain iTSSs in both species. Positional orthologous TSSs were identified for single-copy orthologs by pairwise alignments of the TSS loci (see methods). The proportion of aTSSs that are identified as positional orthologs between the two species is significantly higher than the proportion of positional orthologs in iTSSs or gTSSs (*p* < 0.05, using Fisher's exact test and FDR; Table 1; Additional file 3: Table S2). Hence, aTSSs tend to be more conserved than gTSSs and iTSSs. The proportion of orthologous TSSs is positively correlated with protein sequence similarity for all TSS classes (α = 0.05, using χ^2^-test for linear trend). Thus, orthologous proteins with a higher sequence similarity have a larger fraction of orthologous TSSs.

**Table 1:** Frequency of orthologous TSSs

Class	gTSS	aTSS	iTSS
No. TSS	5112	4082	3382
No. Orthologous genes	2264	1886	1780
Mean TSS per orthologous gene pair	2.26	2.16	1.9
No. Orthologous TSS (%)	1622 (32%)	1556 (38%)	1213 (36%)
No. Lineage specific TSS (%)	3490 (68%)	2526 (62%)	2169 (64%)

TSS loci within a protein-coding gene do not only serve as a binding site for the transcriptional machinery, but are also translated into a protein. We hypothesize that the DNA sequence at TSS loci is more conserved than the amino acid sequence of the gene in which it is found. To test this hypothesis, we compared the sequence similarity of TSS loci (±35 bp around a TSS) with the corresponding orthologous protein sequence similarity. The results show that orthologous aTSS- and iTSS loci are indeed more conserved than the associated orthologs (*p* < 0.05 using Wilcoxon ﻿signed-rank﻿ test; Additional file 4: Table S3). In contrary, gTSS loci were not found to be more conserved than the downstream protein coding gene. Additionally, TSS loci of lineage-specific TSSs are significantly less conserved than the associated orthologous protein sequences (Additional file 4: Table S3).

To quantify the level of orthologous TSS conservation beyond the genus level, we inferred orthologous TSSs between *F. muscicola* and *Chlorogloeopsis fritschii*. The results reveal a similar distribution of the main TSS classes as in the intra-generic comparison (see Additional file 1: Supplementary text for more details). Overall, 11% of the *F. muscicola* TSSs have orthologous TSSs in *C. fritschii*. The most conserved TSS class in the inter-generic comparison is gTSS, followed by aTSS and then iTSS.

### Candidate TSSs putatively involved in the true-branching colony morphology

**Fig. 3:**
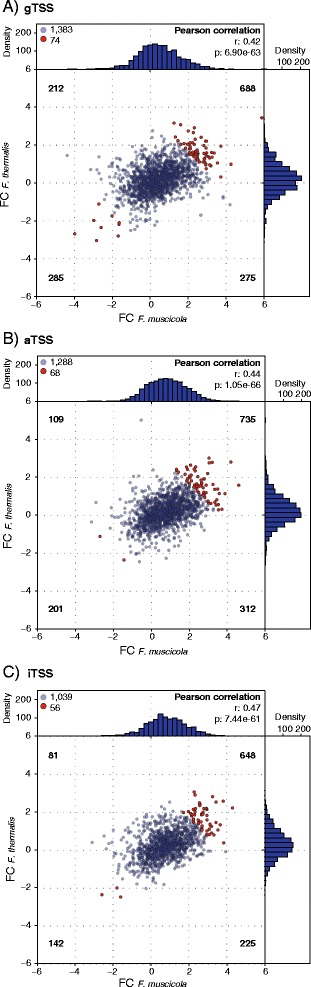
Correlated transcript fold change between orthologous TSSs in *F. muscicola* and *F. thermalis.* Scatterplots of transcript fold changes (FC) for orthologous gTSSs (**a**), orthologous aTSSs (**b**) and orthologous iTSSs (**c**) between *F. muscicola* and *F. thermalis*. Each dot represents an orthologous TSS for which a read coverage information in both conditions was obtained. Dots located in the first quadrant (upper right) are putative transcripts that are up-regulated within the branchless phenotype in both species. Dots located in the third quadrant (lower left) are down-regulated within the branchless phenotype in both species. Orthologous TSSs that do not show a consistent expression fold change are located in the second or fourth quadrant (upper left and lower right, respectively). The total amount of TSSs for each quadrant is listed in the plot corners. The consistent TSSs are the 5% most extreme fold changes and are highlighted as red dots. Transcript fold change is calculated as log2 of the ratio of branchless to true-branching transcription level

The phenotypic similarity of the filamentous, branchless cultures of the two *Fischerella* species already indicates that regulatory plasticity of orthologous genes is likely playing a role in the formation of the branchless phenotype. Here we use the evolutionary comparison between the two *Fischerella* species to identify conserved orthologous TSSs whose transcription fold change in the branchless versus the true-branching phenotype is consistent. Indeed, our results reveal that the ratio of branchless to true-branching transcription level (i.e., transcription fold change) is significantly positively correlated among *Fischerella* orthologous TSSs in the three TSS classes (Fig. 3). To identify orthologous TSSs whose transcription fold change is extreme and consistent between the two *Fischerella* species, we applied a threshold of 5% to the total transcription fold change ratio distribution (Fig. 3; Additional file 5: Table S4). TSSs in this set are putatively involved in the determination of filament morphology.

**Fig. 4:**
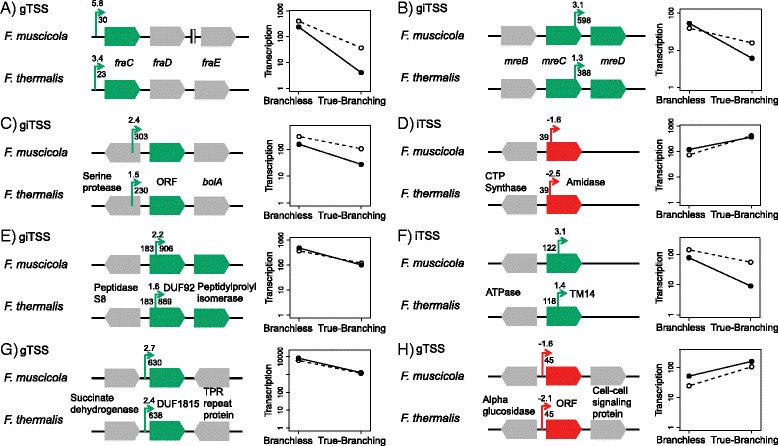
Genetic context of candidate gene examples. (**a**-**h**) Arrows designate identified orthologous TSSs. The transcription fold change (as in Fig. 3) is noted above the arrow. Arrow shade corresponds to the condition in which the TSS was upregulated: branchless (green) or true-branching (red). Distance to the next upstream and downstream 5′ ORF is noted to the left and right of the arrow respectively. ORFs for which the TSS was observed are shaded according to the condition where the TSS was upregulated: branchless (green) or true-branching (red). Neighbouring ORFs are coloured in grey. The genomic context is accompanied by a reaction norm where the phenotype (y-axis) is the TSS transcription level of *F. muscicola* (full circles) and *F. thermalis* (empty circles) in the different environments that induce the branchless and true-branching phenotypes (x-axis). Gene identifier of hypothetical ORFs: **c**) WP_009456078.1 (the amino acid sequence is identical between the two *Fischerella* species) **d**) *F. muscicola*: WP_016867660.1, *F. thermalis*: WP_016870793.1; **e**) *F. muscicola*: WP_016869437.1, *F. thermalis*: WP_009455441.1; **f**) *F. muscicola*: WP_016867107.1, *F. thermalis*: WP_009460349.1; **g**) *F. muscicola*: WP_016865184.1, *F. thermalis*: WP_009456028.1; **h**) *F. muscicola*: WP_016867004.1, *F. thermalis*: WP_016871273.1

The orthologous gTSS with the highest transcription fold change in the branchless phenotype is located upstream of *fraC*. The transcription level reaction norm demonstrates that the elevated transcription level of that gTSS in the branchless phenotype in comparison to the true-branching phenotype is consistent between the two *Fischerella* species (Fig. 4a; Additional file 5: Table S4). Notably, FraC has been previously studied with regards to filament formation in cyanobacteria. In *Anabaena* sp. PCC 7120, *fraC* is encoded in an operon with *fraD* and *fraE* [20]. FraC and FraD have been shown to be involved the in septa formation in *Anabaena* sp. 7120 and have an influence on filament integrity [13, 20]. The *fra* operon is conserved in *F. thermalis* and includes the three *fra* genes as in *Anabaena* sp. PCC 7120 (Fig. 4a). The *fraE* gene in *F. muscicola* is encoded in a different genomic context, yet an orthologous gTSS for *fraE* could be observed in the comparison with *F. thermalis*.

A consistent increased transcription level in the branchless phenotype is observed for an orthologous giTSS that is located within the *mreC* ORF. The giTSS is 598 nt distant from the downstream *mreD* ORF and is hence classified as both genic and intergenic TSS (Fig. 4b; Additional file 5: Table S4). Homologs of MreC and MreD are known to play a role in cell elongation within *E. coli* [21]. Together with MreB, these proteins form an essential membrane bound complex of the elongasome [22]. These three genes are typically encoded within the *mre*-operon that includes *mreB*, *mreC* and *mreD*. The giTSS locus suggests that it serves as an alternative TSS for *mreD* to be expressed independently of the *mre*-operon. A monocistronic expression of *mreD* has been recently documented in the subsection IV cyanobacterium *Fremyella diplosiphon* and it has been suggested that the intergenic region between *mreC* and *mreD* could contain an alternative promoter region [23]. The detection of *fraC* and *mreD* TSSs as putatively involved in the morphology transformation serves as an internal validation for our approach.

In the following we present selected orthologous TSSs for which a highly consistent transcription fold change has been observed and that are associated with genes that encode unknown proteins. A consistently upregulated orthologous gaTSS is observed upstream of a 203 amino acids long hypothetical ORF that is located upstream of a *bolA* homolog encoded at the same orientation (Refseq: WP_009456078.1; Fig. 4c; Additional file 5: Table S4). The amino acid sequence encoded by this ORF is 100% conserved between the two *Fischerella* species and it contains a putative N-terminal transmembrane domain. BolA is known as a transcriptional regulator of *mreB* that is involved in cell elongation [24, 25]. The role of BolA as a determinant of cell shape has been exemplified also in the cyanobacterium *F. diplosiphon*. During photo-acclimation of *F. diplosiphon*, the expression of *bolA* is correlated with *mreB* repression and spherical growth [23, 26]. Homologs of this hypothetical ORF are found in all subsection IV and V sequenced genomes and are always encoded upstream of the *bolA* homolog. The conserved order of these two ORFs raises the possibility that they are transcribed as an operon.

Our analysis reveals that TSSs with a consistent negative transcription fold change, i.e., where the transcription level is lower in the branchless compared to the true-branching phenotype, are generally rare (Fig. 3). A consistent negative transcription fold change was observed for an iTSS located 39 nt downstream of a 5′ ATG of an AmiC-homologous ORF (Fig. 4d, Additional file 5: Table S4). The ORF is 594 amino acids long and it contains a peptidoglycan amino hydrolase (amidase) conserved domain. A Pribnow box motif 5′-TAxxxT-3′ is found 7–13 nt upstream of the iTSS in both *Fischerella* species, yet, no evidence for a truncated ORF was found (i.e., a start codon). Hence, it is possible that this iTSS results in a small RNA rather than an mRNA. Proteins that include an amidase domain may be important for cell septation and separation and are typically localized in the septa hence they have been recognized as relevant for filament morphology in cyanobacteria [27]. A phylogeny of AmiC-homologs in subsection IV and V cyanobacteria indicates that the amidase homolog for which we detected an iTSS is a paralog of *amiC2* (Additional file 1: Figure S5). AmiC2 has been recognized as a component of the septosome and its knockout in *Nostoc punctiforme* leads to clustered cell growth rather then linear filaments that are characteristic to *N. punctiforme* wild type [28].

A giTSS with a consistently higher transcription level in the branchless phenotype is located within a single copy ORF (264 amino acids), which contains a conserved DUF92 transmembrane family domain comprising five transmembrane domains (Fig. 4e; Additional file 5: Table S4). A Pribnow box motif 5′-TAxxxA-3′ is found 8–14 nt upstream of the giTSS in both *Fischerella* species and an in-frame ATG is located 40 nt downstream of the giTSS. Hence, this giTSS could result in an mRNA of a truncated ORF that contains the DUF92 domain. The ORF has homologs in most cyanobacteria as well as in plants and algae. The latter suggests that it is an ancient gene within the phylum and it was probably acquired in the Archaeplastida from the cyanobacterial plastid ancestor.

An iTSS with a consistently higher transcription level in the branchless phenotype is found within a single copy ORF (109 amino acids) that contains a conserved transmembrane-14 family domain (Fig. 4f; Additional file 5: Table S4). A Pribnow box motif 5′-TAxxxT-3′ is located 11–17 nt downstream (in *F. muscicola*; 7–13 nt downstream in *F. thermalis*) but no alternative ORF could be identified. Hence, it is likely that this iTSS results in an sRNA. The ORF has homologs mainly in filamentous cyanobacteria and no homologs have been found outside the phylum.

A gTSS with a consistently higher transcription level in the branchless phenotype is located upstream of a 114 amino acids hypothetical ORF that contains a conserved DUF1815 domain (Fig. 4g; Additional file 5: Table S4). This ORF has homologs in most cyanobacteria, including filamentous and unicellular organisms. No homologs were found outside cyanobacteria suggesting that this ORF is specific to the phylum.

Another gTSS with a consistently lower transcription level in the branchless phenotype is found upstream of a hypothetical ORF (200 amino acids) (Fig. 4h; Additional file 5: Table S4). The ORF contains a single transmembrane domain and a coiled-coil domain; it has homologs in most cyanobacteria and has no homologs outside the phylum.

In summary, we find that many of the TSSs having consistent transcription fold-changes are involved in the transcription of unknown proteins whose distribution is not specific to true-branching cyanobacteria, yet they are unique to the cyanobacterial phylum.

## Discussion

Here we provide evidence for the importance of the ‘plasticity first’ hypothesis for the evolution of the trait ‘true-branching’ in cyanobacteria. Our study uncovers a plastic branchless phenotype in the true-branching *Fischerella* species that is induced in the wildtype genotype under specific environmental conditions (i.e., supplementation with sucrose). For investigating cyanobacteria morphological diversity a similar approach has been undertaken in *Nostoc linckia*. This species, which is characterized by linear filaments, has been demonstrated to form branching filaments after ultraviolet irradiation treatment [29]. Further studies on the genetic underpinnings of filament formation used transposon mutagenesis in the unicellular cyanobacterium *Synechococcus elongatus* PCC 7942 to induce filaments [30]. We here present a novel approach that combines the dRNA-seq method with comparative genomics of two species having a similar phenotypic response to the same environmental change. The genomic comparison enabled the specification of orthologous TSSs for orthologous genes. Orthologous TSSs that are consistently regulated in the two species serve as an indication for the evolutionary conservation of transcriptional regulatory elements.

Our analysis reveals that positional orthologous TSSs of orthologous genes within the *Fischerella* species are more frequent than in the *F. muscicola* and *C. fritschii* comparison. This finding is in agreement with earlier observations that TSS conservation is negatively correlated with evolutionary distance in bacteria [31]. Moreover, the proportion of orthologous gTSSs within the *Fischerella* genus is positively correlated with the orthologous protein sequence similarity. Hence, conserved orthologous genes within the genus are expected to be transcribed from conserved orthologous TSSs.

The intragenic comparison reveals that orthologous aTSSs are significantly enriched within single copy orthologous genes in comparison to iTSSs and gTSSs. A recent large-scale comparative transcriptomic study showed that the frequency of antisense transcription is exponentially correlated with the genome AT-content [32]. This result led to the suggestion that antisense transcription is mainly a consequence of transcriptional noise due to stochastic transcription factor binding to spurious promoters throughout the genome [32]. Indeed, cyanobacterial genomes, including the ones analysed here, are commonly AT-rich and several studies of transcriptional regulation in cyanobacteria reported a high frequency of antisense transcription (e.g. [33, 34]). Hypothetically, sequence conservation of protein coding genes in AT-rich genomes could preserve promoter-like sites within genes and TSSs resulting in non-functional transcripts (i.e., transcriptional noise) [31]. However, this would imply that iTSSs and aTSSs would be equally conserved, which is not the case in our analysis. This suggests that some of the aTSSs, especially those that are conserved between the *Fischerella* strains, are indeed functional.

In our study, we made use of the phenotypic similarity among the two sister species and only searched for orthologous TSSs that were consistently regulated as they most likely serve as an indication for the evolutionary conservation of the transcriptional regulatory elements causative for the true-branching phenotype. We found the transcription fold change of the orthologous genes to be significantly positively correlated between the two *Fischerella* species, and detected consistent transcription fold changes of several genes encoding proteins that are known to play a role in filament formation and integrity, e.g. *fraC*. Most of the TSSs with consistent transcription fold change in *Fischerella* are found in genes that are not specific to true-branching cyanobacteria. This supports our hypothesis that the true-branching morphology observed in Stigonematales is not dependent on a genus-specific gene gain or loss; rather it is the result of genus-specific transcriptional regulation.

## Conclusions

The phylogenetic reconstruction of cyanobacterial phylogeny shows that the true-branching Stigonematales form a monophyletic clade within the Nostocales clade [10, 11], indicating that the ancestor of true-branching cyanobacteria was phenotypically plastic. Due to variation in the population on the transcriptional level and dependent on the environmental stimulus, the ancestor must have had the potential to form either linear or true-branching filaments. During the evolution of Stigonematales, the true-branching morphology became fixed, i.e., constitutively expressed, so that its formation no longer depended on the inducing environment. Our results suggest that these changes may have occurred in the regulatory regions. Through exposure to a novel environmental condition, we were able to generate non-branching, linear filaments in *Fischerella*, akin to the ancestral phenotype and so exposed remaining hidden variation (i.e., plasticity) in filament morphology. We propose that the evolution of the true-branching morphology happened via genetic accommodation, where the phenotypic plasticity of an initially environmentally induced trait decreases to an extend to where that trait is fixed [3, 4].

While genuine cell differentiation is rare in the prokaryotic domain, phenotypic plasticity is rather common. Transient alteration between unicellular and filamentous forms have been observed in organisms that are commonly referred as unicellular, such as *Escherichia coli* and *Pseudomonas aeruginosa* [35]. Nonetheless, the role of phenotypic plasticity in the emergence of novel traits has been so far overlooked. Phenotypic heterogeneity has been observed to play a role in microbial evolution [36]. Our study demonstrates how the fixation of a plastic phenotype can lead to the evolution of the true-branching trait in cyanobacteria.

## Methods

### Strains and culturing conditions

*Fischerella muscicola* PCC 7414, *Fischerella thermalis* PCC 7521 and *Chlorogloeopsis fritschii* PCC 6912 were obtained from the Pasteur culture collection of Cyanobacteria (PCC), France. Stock cultures were grown photoautotrophically at a 12 h/12 h light/dark regime in liquid BG11 or BG11o medium [9], at 37 °C and a light intensity of 30 μmol/m^2^ s^1^. To identify environmental conditions that induce the formation of homogeneous filament morphology (e.g., branchless filaments), we carried out preliminary experiments in which we tested the effect of supplementing sucrose or NaCl in various concentrations to BG11 and BG11o grown cultures. Using this approach, we minimize cell type heterogeneity in the culture towards the objective of capturing a genuine transcription profile of specific morphotypes. To this end, cultures were grown for 30 days in BG11 and BG11o in each of the following conditions: for photoheterotrophic growth, supplemented with different concentrations of sucrose (0, 10, 100 mM); for photoautotrophic growth, supplemented with NaCl (0, 50, 100, 250, 450, 600 mM)﻿; and for heterotrophic growth, plated on solid media (1% agarose) supplemented with sucrose (10, 100 mM) and incubated in darkness. Morphological changes were documented by microscopy examination every 2–3 days. Environmental conditions that were found to induce a homogeneous morphotype were reproduced and three replicate samples of each morphotype were selected for sequencing.

### RNA isolation

Samples for RNAseq were harvested by filtration with 8 μm nitrocellulose filters, subsequently resuspended in RNA lysis reagent (Invitrogen), rapidly frozen in liquid nitrogen and stored at −80 °C until use. Total RNA was isolated using Concert Plant RNA Reagent (Invitrogen) as previously described [37] with few modifications. Briefly, frozen samples were thawed on ice, and cells were disrupted 6 × 30 s with 212–300 μm diameter acid-washed glass beads in a tissue lyser (SpeedMill, Analytik Jena). The supernatant was separated from the glass beads and cell debris by centrifugation (10 min, 12,000 *x g*, 4 °C). DNA digestion was performed after RNA isolation with RNase-free DNase (Thermo Scientific) for one hour at 37 °C. RNA integrity was checked on RNA nano-chips using an Agilent Bioanalyzer 2100. Equal amounts of RNA from the three biological replicates were pooled for sequencing.

### 5′-dRNA-seq

The cDNA libraries were prepared by Vertis Biotechnologie AG, Germany (http://www.vertis-biotech.com/) and sequenced with Illumina HiSeq2000 (50 bp read length). For discrimination between processed RNA (RNAs with a 5′-P) and primary transcripts (RNAs with a 5′-PPP), we followed the dRNA-seq approach described in [15]. Briefly, one half of each RNA sample was enriched for primary transcripts by treatment with terminator-5′-phosphate-dependent exonuclease (TEX, Epicentre). TEX(−) samples were treated with the *RiboZero rRNA Removal Kit* (Epicentre) for rRNA depletion. For strand-specific sequencing, both TEX(+)/(−) samples were treated with tobacco acid pyrophosphatase (TAP) to convert 5′-PPP to 5′-P and a 3′-poly-A was ligated, followed by RNA adapter primer ligation to the 5′-P and cDNA synthesis.

### Transcription start sites (TSSs) inference

The 3′-poly-A ligation step within the library construction might lead to a bias of A in the 3′ end or a bias of T in the 5′ end of reads. Therefore, every read was clipped towards a 3′-poly-A or 5′-poly-T that exceeded the length of a maximum poly-A or poly-T, observed in the genomes. Only reads with a minimum length of 20 nucleotides after clipping were kept. The remaining reads were mapped onto the reference genomes [11] using blastall with the option blastn [38] (query filtering parameter false, maximum e-value 10^−4^). A read was defined as mapped, if the alignment length is at least 80% of the read length, with a maximum of 4 mismatches and no gaps. Reads that mapped onto rDNA operonic regions or with multiple best scoring regions were discarded. Read coverage for each genomic position and dRNA-seq sample was normalized by the size of the smallest dRNA-seq sample. TSS prediction was performed with TSSpredator (v1.0.4) [18], applying a clustering distance of 30 bp. TSSs detected in one species and in any condition were clustered again using a sliding window size of 35 bp and TSSs with the highest step heights within the resulting clusters were kept. TSSs were classified depending on the location relative to an ORF. Genic TSSs (gTSSs) were found in a 1000 nt distance upstream of an ORF on the same strand. The 5′-untranslated region (5’UTR) threshold was determined from the mean intergenic distance within each species (*F. muscicola*: 1168 bp; *F. thermalis*: 1243 bp; *C. fritschii*: 1162 bp). Internal (iTSSs) and antisense TSSs (aTSS) were found within an ORF encoded on the same or complementary strand, respectively. TSSs that could not be classified into at least one category were classified as intergenic (nTSS). The transcription fold change was calculated as the logarithm to the base 2 of the ratio of TSS transcript level in the branchless and the true-branching sample. Only TSSs that have a transcript level > 0 in both conditions were considered.

### Single copy orthologs detection

A reciprocal best BLAST hit procedure was performed on a database containing the proteomes of the three species. Hits were found with blastall and the blastp option from an all -against- all BLAST by considering a minimum of 30% identity, maximum e-value of 10^−10^ and parameters optimized for orthology detection (−F “m S” –s T) [39]. Reciprocal best BLAST hits were aligned globally with needle [40] to filter out pairs with less than 30% global identity. The remaining pairs were used for protein families reconstruction with mcl [41]. Single copy orthologs for a set of species are clusters containing exactly one gene from each of the species. Conserved domains where identified using the conserved domains database tool [42].

### Comparative TSS analysis

For the gTSS conservation analysis, UTR alignments were generated from 5′-UTR regions (1000 nt) of single copy orthologs. TSSs were relocated in the alignments and classified as “ortholog”, if they were separated by not more than 35 bp from each other. For the aTSS and iTSS conservation analysis, alignments of the single copy orthologous protein sequences were transformed into codon alignments and classified as “ortholog” by using the same distance criterion. All TSSs that did not pass the distance criterion were classified as “singleton”. Orthologous TSS loci were defined as regions ±35 bp around each TSS. Genes with candidate orthologous TSSs were screened for transmembrane domains using TMHMM (v2.0) [43]. Proteins with transmembrane domains were screened for signal peptides, using SignalP (v4.1) [44].

## Additional files

Additional file 1: Supplementary text and Supplementary figures. **Figure S1.** Additional Phenotypes observed in *F. muscicola* and *F. thermalis*. **Figure S2.** Nucleotide composition of TSS upstream regions. **Figure S3.** TSS location relative to the ORF. **Figure S4.** Distribution of TSS classes in ORFs. **Figure S5.** Amidase phylogenetic tree. **Figure S6.** Phenotypic plasticity in colony morphology in *Chlorogloeopsis fritschii* PCC 6912. (PDF 11443 kb)

Additional file 2: Table S1. TSS statistics. (PDF 54 kb)

Additional file 3: Table S2. Orthologous TSS conservation. For each pair of TSS-classes we compare the ratio of orthologous TSSs to the total frequency of TSSs (using Fisher's exact test). The class with a larger ratio includes more positional orthologs and is therefore more conserved. TSSs that fit into more than one class (i.e., gaTSS, giTSS, aiTSS and gaiTSS) were excluded from the analysis. (PDF 52 kb)

Additional file 4: Table S3. TSS-locus sequence similarity in comparison to ORF sequence similarity. (PDF 55 kb)

Additional file 5: Table S4. Candidate ORFs involved in true-branching phenotype. The table includes all ORFs in which one or more consistent orthologous TSSs have been detected. Consistent TSSs (as in Fig. 3) are written in red, orthologous TSSs are written in blue and singelton TSSs are written in gray. ORF and TSS positions on the left are according to Dagan et al. 2013 [11] annotation. ORF and TSS positions according to RefSeq genome annotation are listed on the right. (XLSX 318 kb)

## Abbreviations

aTSS: Antisense TSS
dRNA-seq: Differential RNA sequencing
gTSS: Genic TSS
HRF: Hormogonium-repressing-factor
iTSS: Internal TSS
mRNA: Messenger RNA
nTSS: Intergenic TSS
ORF: Open reading frame
PCC: Pasteur Culture collection of Cyanobacteria
RNA: Ribonucleic acid
TEX: Terminator exonuclease
TSS: Transcription start site
UTR: Untranslated regulatory region
