# Supplementary material for: Plasticity first: molecular signatures of a complex morphological trait in filamentous cyanobacteria
Source: BMC Evol Biol. 2017 Aug 31;17:209. doi: 10.1186/s12862-017-1053-5 (PMC5580265; doi:10.1186/s12862-017-1053-5)
Supplement: Supplementary file 1 — Supplementary text and Supplementary figures. Figure S1. Additional Phenotypes observed in F. muscicola and F. thermalis. Figure S2. Nucleotide composition of TSS upstream regions. Figure S3. TSS location relative to the ORF. Figure S4. Distribution of TSS classes in ORFs. Figure S5. Amidase phylogenetic tree. Figure S6. Phenotypic plasticity in colony morphology in Chlorogloeopsis fritschii PCC 6912. (PDF 11443 kb) [file 12862_2017_1053_MOESM1_ESM.pdf]

Plasticity first: molecular signatures of a complex morphological trait in filamentous cyanobacteria

Robin Koch, Anne Kupczok, Karina Stucken, Judith Ilhan, Katrin Hammerschmidt, Tal Dagan

Supplementary material

Supplementary text

Phenotypic plasticity of the multiseriate colony morphology

For the generation of the ancestral-like, aseriate phenotype, we cultured the multiseriate *C. fritschii* PCC 6912 under different environmental conditions, such as photoheterotrophic growth (0, 10 and 100 mM sucrose), heterotrophic growth on solid media (darkness and 100 mM sucrose), high temperature (45°C), growth on solid media, and NaCl supplement (50, 100 and 250 mM). Multiseriate filaments or aseriate aggregations were achieved by either increasing the salt concentration, or by shifting the cultures from heterotrophic growth (darkness) to photoheterotrophic growth on solid medium. To identify genes whose transcriptional regulation correlate with the multiseriate phenotype, we compared the transcriptome of the wild-type *Chlorogloeopsis* culture to the NaCl supplemented culture in which the cells show aseriate cell clusters instead of multi-seriate filaments (Fig. S6).

The primary transcriptome of *C. fritschii*

RNA samples of *Chlorogloeopsis fritschii* were processed as described for the two *Fischerella* species. The TSS-class distribution of *C. fritschii* is similar compared to the ones of *Fischerella*. A total of 19,420 TSSs distributes as follows: gTSS: 4,084 (21%), aTSS: 5,217 (27%), iTSS: 3,431 (18%), gaTSS: 2,008 (10%), giTSS: 3,125 (16%), gaiTSS: 1,516 (8%). The proportions of nTSS and aiTSS is <1%. The proportion of constitutive TSSs observed in the multiseriate and aseriate phenotypes is 42% in *C. fritschii* (Table S1A). The untranslated regulatory region (UTR) length distribution is similar among the three species (Fig. S3).

TSSs were recovered for 89% of the ORFs in *C. fritschii*. Also here, for the majority of ORFs, TSSs of more than one TSS-class have been detected (Fig. S4). The median

TSS-frequency per ORF is 1 for most TSS classes (Table S1B). The ORF length and the frequency of TSSs per ORF are correlated in all three species for aTSSs and iTSSs (Table S1B).

Evolutionary conservation of transcriptional start sites in orthologous genes – comparison between *F. muscicola* and *C. fritschii*

The intergeneric comparison between *F. muscicola* and *C. fritschii* reveals a similar distribution of the main TSS-classes as for the intrageneric comparison, including 2,697 orthologous gene pairs. The proportion of positional orthologous TSSs between *F. muscicola* and *C. fritschii* is lower than in the intragenic comparison (Table S2). The proportion of gTSSs having positional orthologs in both species is higher than the proportion of positional orthologs in the other two TSS-classes ($p<0.05$, using Fisher test and FDR; Table S2). Additionally, a significantly higher proportion of positional orthologs was observed in the aTSS-class in comparison to the iTSS class ($p<0.05$, using Fisher test and FDR; Table S2). Hence, in the intergeneric comparison, the most conserved TSS-class is gTSS, followed by aTSS and then iTSS.

No significant differences at the level of protein sequence similarity and the similarity of orthologous aTSS- or iTSS-loci were detected. Furthermore, TSS-loci of singleton TSSs are significantly less conserved than the associated orthologous protein sequence in all comparisons (Table S3).

Candidate TSSs putatively involved in the complex colony morphology trait of *C. fritschii*

The comparison of orthologous TSSs between *F. muscicola* and *C. fritschii* revealed no significant correlation in the transcription fold change of gTSS and aTSS and a weak correlation for the iTSS (r=0.15, p=0.003).

Supplementary Figures

Figure S1: Additional Phenotypes observed in *F. muscicola* and *F. thermalis.*

A) *F. muscicola* PCC 7414
BG11 50mM NaCl

B) *F. thermalis* PCC 7521
BG11o 100mM NaCl

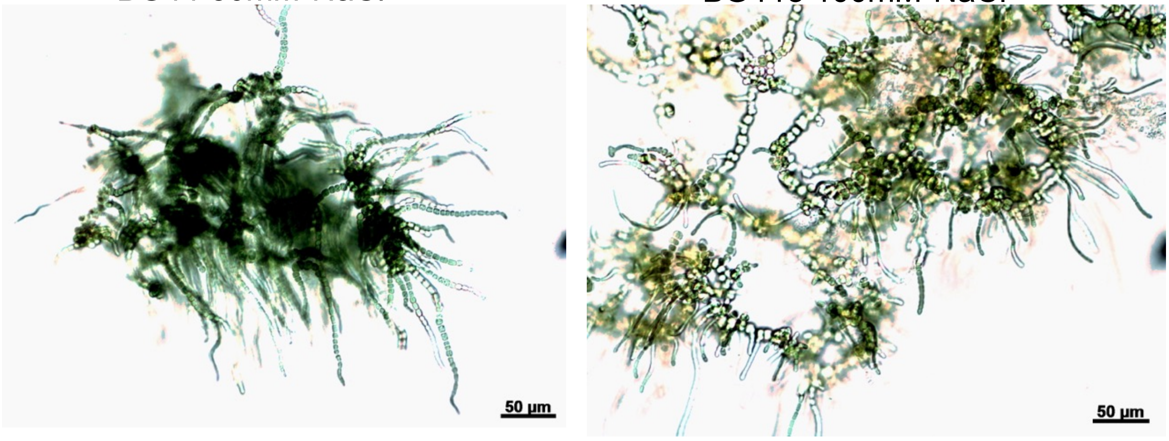

Figure S2: Nucleotide composition of TSS upstream regions

Nucleotide frequency within a 50 bp window upstream of a TSS were calculated. Negative values on the x-axis reflect the distance of the position relative to the TSS (TSS at +1). In every TSS class and species, an enrichment for the nucleotides adenine and thymine within the -11 to -5 region can be observed, indicating the presence of a putative -10 box (Pribnow box). A motif search by using the motif enrichment tool MEME with a fixed window size of 45 nt was performed. The sequence logo created from MEME and the amount of occurrences after prediction confirm the nucleotide composition study and support the presence of a general or extended -10 box upstream of most TSSs.

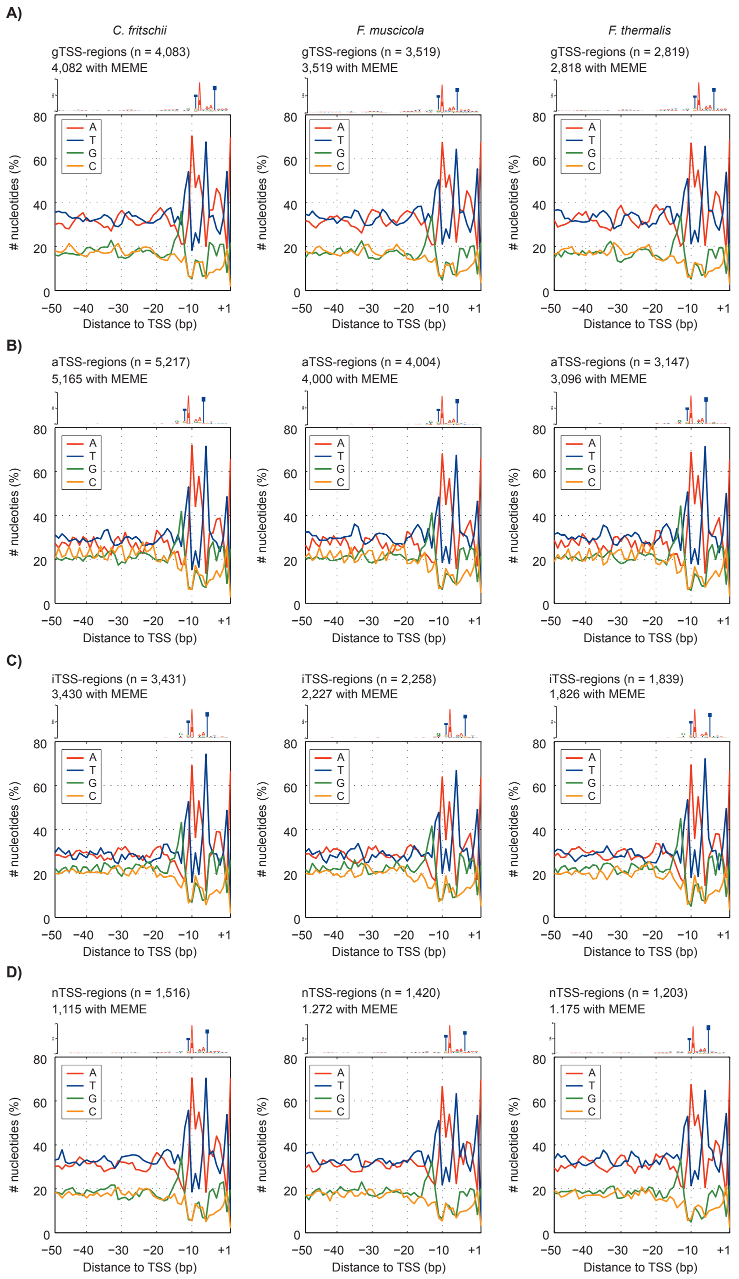

Figure S3: TSS location relative to the ORF.

(A) UTR lengths. gTSSs shows smaller UTR lengths than the giTSSs or the gaTSSs and giTSSs show smaller UTR lengths than gaTSSs. Mean UTR length is 374 bp in *F. muscicola*, 377 bp in *F. thermalis* and 385 bp in *C. fritschii.* (B) TSS locations within an ORF on the opposite strand. aTSSs tend to be located on the 3'-end of ORFs compared to gaTSSs. (C) TSS location within an ORF on the same strand. iTSSs tend to be located closer to the 5'-end of ORFs compared to giTSSs. CDF: cumulative distribution function.

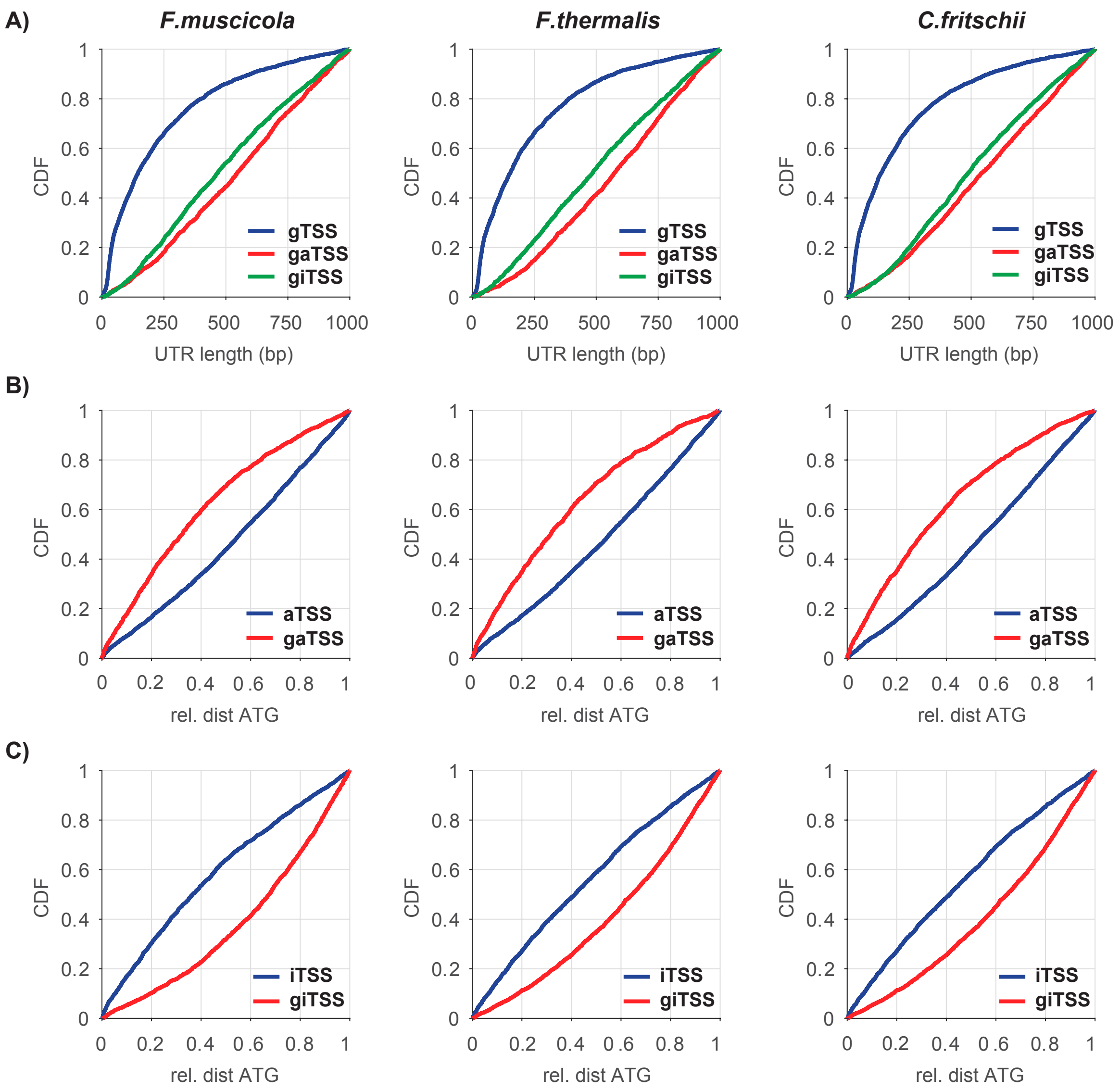

Figure S4: Distribution of TSS classes in ORFs.

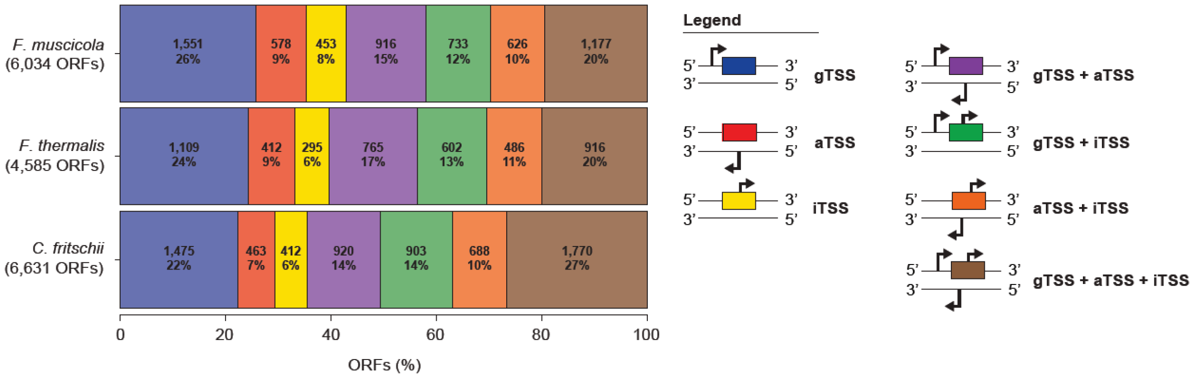

Figure S5: Amidase phylogenetic tree. Amino acid sequences were aligned using MAFFT (1) and a phylogenetic tree was reconstructed using PhyML (2) with 100 bootstrap replicates (support values shown for all internal nodes).

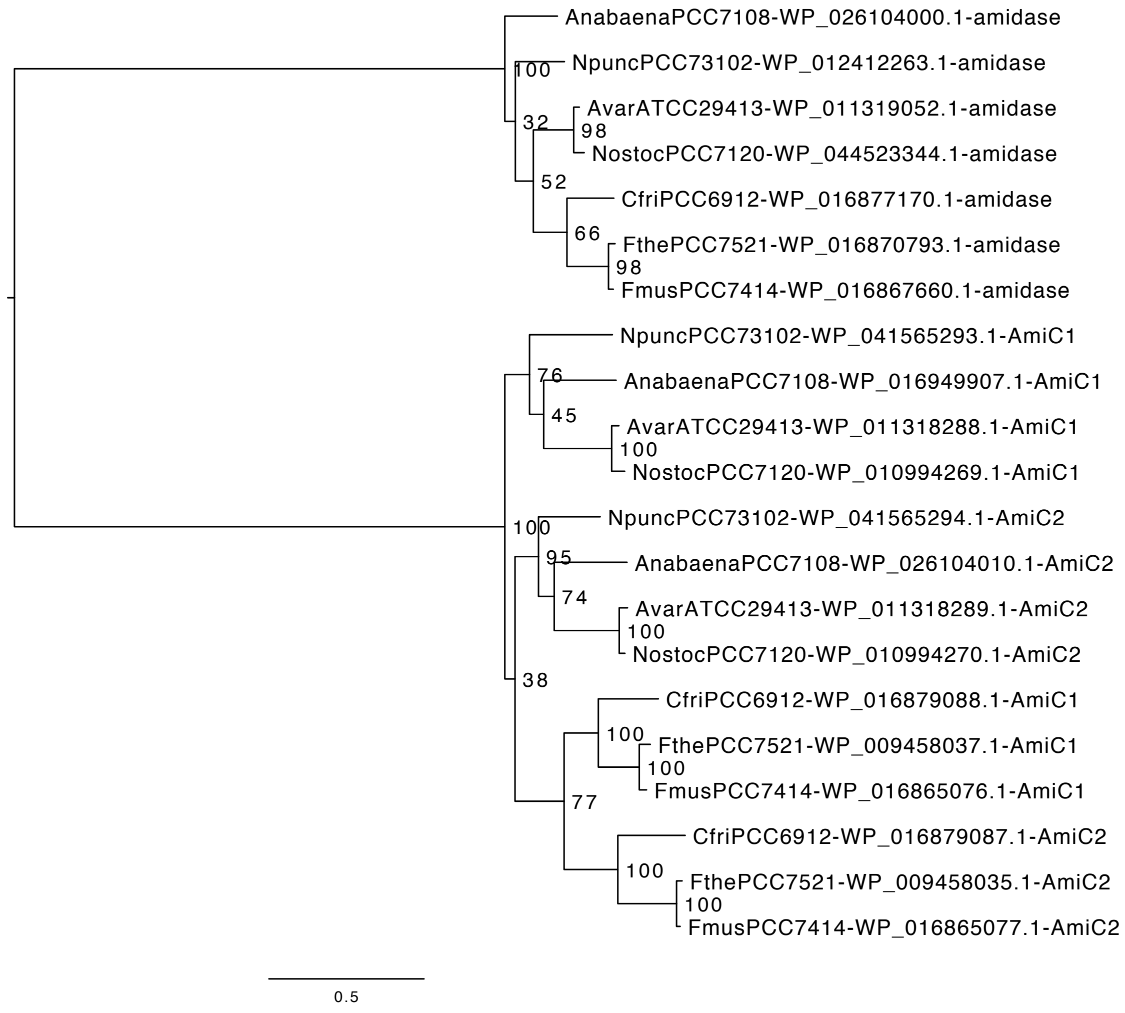

Figure S6: Phenotypic plasticity in colony morphology in *Chlorogloeopsis fritschii* PCC 6912

(A) When grown in BG11, *C. fritschii* developed multi-seriate filaments. (B) Culture grown with sodium chloride (100 mM NaCl) resulted in aseriate cell clusters. (C) *C. fritschii* grown on plates supplemented with sucrose (100 mM).

A)

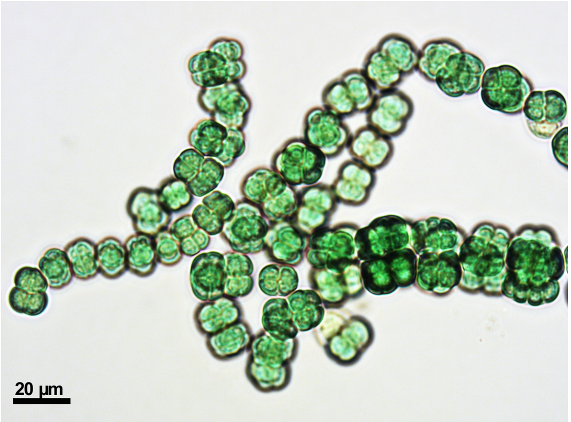

B)

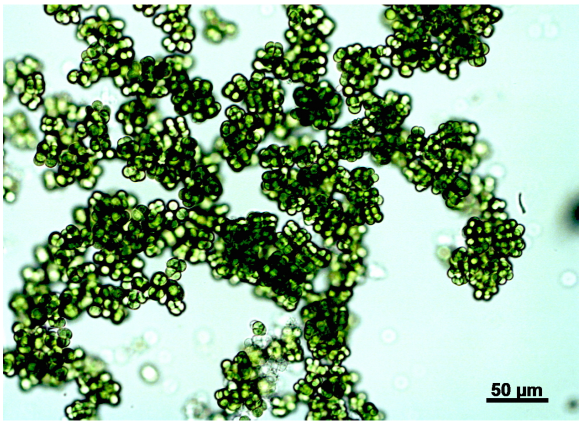

C)

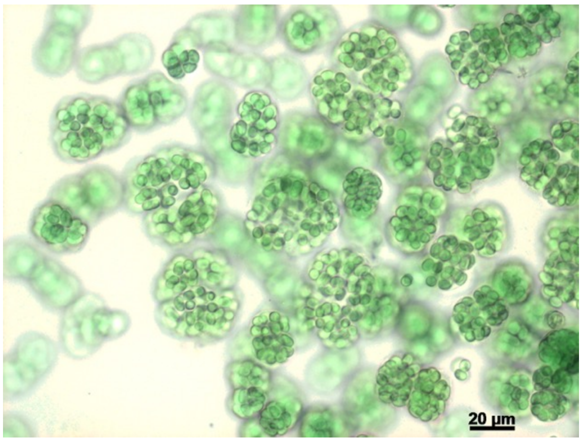

1. Katoh K, Standley DM. MAFFT multiple sequence alignment software version 7: improvements in performance and usability. Mol Biol Evol. 2013;30:772–780.

2. Guindon S, et al. New algorithms and methods to estimate maximum-likelihood phylogenies: assessing the performance of PhyML 3.0. Systematic Biol. 2010;59:307–321.
