# Supplementary material for: Plasticity first: molecular signatures of a complex morphological trait in filamentous cyanobacteria
Source: BMC Evol Biol. 2017 Aug 31;17:209. doi: 10.1186/s12862-017-1053-5 (PMC5580265; doi:10.1186/s12862-017-1053-5)
Supplement: Supplementary file 2 — TSS statistics. (PDF 54 kb) [file 12862_2017_1053_MOESM2_ESM.pdf]

Table S1: TSS statistics.

A) Number of detected TSSs. TSSs are condition specific if they are found as enriched in only one condition by TSSPredator and the step height in the other condition is 0.

	Both conditions	Treatment only	Control only	Total
F. muscicola	13,710 (91%)	971 (6%)	456 (3%)	15,137
F. thermalis	11,442 (96%)	339 (3%)	187 (2%)	11,968
C. fritschii	16,931 (87%)	1,217 (6%)	1,272 (7%)	19,420

B) Frequency of TSS per ORF

***F. muscicola* PCC 7414**

Class	TSSs	ORFs	Min TSS/ORF	Max TSS/ORF	Median TSS/ORF	Mean TSS/ORF	r2*	p*
gTSS	7,427	4,377	1	8	1	1.70	0.002	0.010
aTSS	5,690	3,297	1	8	1	1.73	0.229	1.88E-182
iTSS	4,554	2,989	1	8	1	1.52	0.134	2.93E-95
nTSS	1,420	-	-	-	-	-	-	-

***F. thermalis* PCC 7521**

Class	TSSs	ORFs	Min TSS/ORF	Max TSS/ORF	Median TSS/ORF	Mean TSS/ORF	r2*	p*
gTSS	5,767	3,392	1	7	1	1.70	4.928E-04	0.195
aTSS	4,406	2,579	1	9	1	1.71	0.228	5.81E-147
iTSS	3,566	2,299	1	10	1	1.55	0.108	6.00E-59
nTSS	1,203	-	-	-	-	-	-	-

***C. fritschii* PCC 6912**

Class	# TSS	# ORF	Min TSS/ORF	Max TSS/ORF	Median TSS/ORF	Mean TSS/ORF	r2*	p*
gTSS	9,242	5,068	1	7	2	1.82	0.00	0.0229
aTSS	7,264	3,841	1	18	1	1.89	0.27	5.92E-260
iTSS	6,595	3,773	1	10	1	1.75	0.17	1.35E-158
nTSS	1,516	-	-	-	-	-	-	-

* Spearman correlation test between ORF length and frequency of TSSs
