# Supplementary material for: Plasticity first: molecular signatures of a complex morphological trait in filamentous cyanobacteria
Source: BMC Evol Biol. 2017 Aug 31;17:209. doi: 10.1186/s12862-017-1053-5 (PMC5580265; doi:10.1186/s12862-017-1053-5)
Supplement: Supplementary file 3 — Orthologous TSS conservation. For each pair of TSS-classes we compare the ratio of orthologous TSSs to the total frequency of TSSs (using Fisher's exact test). The class with a larger ratio includes more positional orthologs and is therefore more conserved. TSSs that fit into more than one class (i.e., gaTSS, giTSS, aiTSS and gaiTSS) were excluded from the analysis. (PDF 52 kb) [file 12862_2017_1053_MOESM3_ESM.pdf]

Table S2. Orthologous TSS conservation. For each pair of TSS-classes we compare the ratio of orthologous TSSs to the total frequency of TSSs (using Fisher exact test). The class with a larger ratio includes more positional orthologs and is therefore more conserved. TSSs that fit into more than one class (i.e., gaTSS, giTSS, aiTSS and gaiTSS) were excluded from the analysis.

A) Comparison of *F. muscicola* and *F. thermalis*

TSS-Type	# gTSS	# aTSS	# iTSS
Ortholog	796	994	513
Singleton	1,639	1,786	1,091
Total	2,435	2,780	1,604

	H1: gTSS > aTSS	H1: gTSS < aTSS	H1: gTSS > iTSS	H1: gTSS < iTSS	H1: aTSS > iTSS	H1: aTSS < iTSS
p-value:	0.991	0.011	0.332	0.693	0.006	0.995
FDR corrected:	0.991	**0.032**	0.498	0.995	**0.018**	0.995

Conclusion: aTSS positional orthologs are enriched (i.e., more conserved) in comparison to gTSS and iTSS. No difference between gTSS and iTSS

B) Comparison of *F. muscicola* and *C. fritschii*

TSS-Type	# gTSS	# aTSS	# iTSS
Ortholog	449	314	147
Singleton	2,397	3,422	2,195
Total	2,846	3,736	2,342

	H1: gTSS > aTSS	H1: gTSS < aTSS	H1: gTSS > iTSS	H1: gTSS < iTSS	H1: aTSS > iTSS	H1: aTSS < iTSS
p-value:	2.200E-16	1.000	2.200E-16	1.000	0.001	0.999
FDR corrected:	**3.300E-16**	1.000	**3.300E-16**	1.000	**0.001**	1.000

Conclusion: gTSS positional orthologs are enriched (i.e., more conserved) in comparison to aTSS and iTSS. aTSS positional orthologs are enriched in comparison to iTSS.
