# Supplementary material for: Plasticity first: molecular signatures of a complex morphological trait in filamentous cyanobacteria
Source: BMC Evol Biol. 2017 Aug 31;17:209. doi: 10.1186/s12862-017-1053-5 (PMC5580265; doi:10.1186/s12862-017-1053-5)
Supplement: Supplementary file 4 — TSS-locus sequence similarity in comparison to ORF sequence similarity. (PDF 55 kb) [file 12862_2017_1053_MOESM4_ESM.pdf]

Table S3. TSS-locus sequence similarity in comparion to ORF sequence similarity

A) TSS locus vs. protein sequence similarity, *F. muscicola* / *F. thermalis*

Class	Analysis	TSS-type	Median TSS-locus similarity (%)	Median Protein similarity (%)	Median*	p*	Conclusion*
gTSS	per TSS	Positional Ortholog	96	96	0.46	**1.332E-17**	Protein sequences more similar than TSS-loci
		Singleton	86	95	6.97	**5.914E-278**	Protein sequences more similar than TSS-loci
	per orthologous gene	Positional Ortholog	95	96	0.71	**3.841E-10**	Protein sequences more similar than TSS-loci
		Singleton	86	95	7.67	**1.186E-153**	Protein sequences more similar than TSS-loci
aTSS	per TSS	Positional Ortholog	96	96	-0.59	**5.126E-19**	TSS-loci more similar than protein sequences
		Singleton	94	95	0.49	**3.658E-07**	Protein sequences more similar than TSS-loci
	per orthologous gene	Positional Ortholog	96	96	-0.46	**1.148E-06**	TSS-loci more similar than protein sequences
		Singleton	94	95	0.86	**5.797E-14**	Protein sequences more similar than TSS-loci
iTSS	per TSS	Positional Ortholog	96	96	-0.67	**6.917E-18**	TSS-loci more similar than protein sequences
		Singleton	94	95	0.26	**3.955E-03**	Protein sequences more similar than TSS-loci
	per orthologous gene	Positional Ortholog	96	96	-0.67	**2.393E-08**	TSS-loci more similar than protein sequences
		Singleton	94	95	0.63	**1.274E-07**	Protein sequences more similar than TSS-loci

B) TSS locus vs. protein sequence similarity, *F. muscicola* / *C. fritschii*

Class	Analysis	TSS-type	Median TSS-locus similarity (%)	Median Protein similarity (%)	Median*	p*	Conclusion*
gTSS	per TSS	Positional Ortholog	73	82	8.34	**7.394E-93**	Protein sequences more similar than TSS-loci
		Singleton	51	80	21.80	**0.000E+00**	Protein sequences more similar than TSS-loci
	per orthologous gene	Positional Ortholog	73	82	8.49	**7.621E-41**	Protein sequences more similar than TSS-loci
		Singleton	52	80	21.10	**2.075E-272**	Protein sequences more similar than TSS-loci
aTSS	per TSS	Positional Ortholog	81	81	0.11	6.345E-02	No differences between similarites
		Singleton	79	79	1.43	**2.473E-13**	Protein sequences more similar than TSS-loci
	per orthologous gene	Positional Ortholog	81	81	-0.29	1.425E-01	No differences between similarites
		Singleton	78	79	2.03	**1.428E-14**	Protein sequences more similar than TSS-loci
iTSS	per TSS	Positional Ortholog	81	82	0.24	8.828E-01	No differences between similarites
		Singleton	79	79	0.83	**7.625E-04**	Protein sequences more similar than TSS-loci
	per orthologous gene	Positional Ortholog	81	82	0.46	9.225E-01	No differences between similarites
		Singleton	79	79	1.28	**1.661E-06**	Protein sequences more similar than TSS-loci

(*) Using Wilcoxon ranksum test
